# Modulation of Inherent Niches in 3D Multicellular MSC Spheroids Reconfigures Metabolism and Enhances Therapeutic Potential

**DOI:** 10.3390/cells10102747

**Published:** 2021-10-14

**Authors:** Li-Chi Chen, Hsin-Wen Wang, Chieh-Cheng Huang

**Affiliations:** Institute of Biomedical Engineering, National Tsing Hua University, Hsinchu 30013, Taiwan; richard21708@gmail.com (L.-C.C.); rosycloud.w@gapp.nthu.edu.tw (H.-W.W.)

**Keywords:** mesenchymal stem cells, cell therapy, 3D cell spheroids, immunomodulation, metabolic reconfiguration

## Abstract

Multicellular spheroids show three-dimensional (3D) organization with extensive cell–cell and cell–extracellular matrix interactions. Owing to their native tissue-mimicking characteristics, mesenchymal stem cell (MSC) spheroids are considered promising as implantable therapeutics for stem cell therapy. Herein, we aim to further enhance their therapeutic potential by tuning the cultivation parameters and thus the inherent niche of 3D MSC spheroids. Significantly increased expression of multiple pro-regenerative paracrine signaling molecules and immunomodulatory factors by MSCs was observed after optimizing the conditions for spheroid culture. Moreover, these alterations in cellular behaviors may be associated with not only the hypoxic niche developed in the spheroid core but also with the metabolic reconfiguration of MSCs. The present study provides efficient methods for manipulating the therapeutic capacity of 3D MSC spheroids, thus laying solid foundations for future development and clinical application of spheroid-based MSC therapy for regenerative medicine.

## 1. Introduction

Mesenchymal stem cells (MSCs) have been widely explored for their broad-ranging potential in regenerative medicine, especially in cell therapy [1]. Initially, the therapeutic benefits of MSC transplantation were believed to be attributed to the replenishment of the required cell types through MSC differentiation [2,3]. However, accumulating evidence indicates that it is the secretome of MSCs exhibit the major and diverse therapeutic functions, including anti-apoptosis, pro-angiogenesis, and immunomodulatory effects [1]. By secreting a broad spectrum of bioactive molecules in free forms or encapsulated in extracellular vesicles, the administered MSCs can establish a pro-regenerative microenvironment to promote tissue repair.

Nevertheless, the effectiveness of MSC-based cell therapies in clinical trials remains controversial. It has been reported that transplanted MSCs may not be able to survive under the inhospitable environments of injured/diseased tissues for a prolonged period, thereby limiting the ultimate therapeutic outcomes. As a result, MSCs must be carefully tuned prior to transplantation to achieve the desired curative efficacy. Strategies that can benefit the post-engrafted survival of MSCs and their paracrine potential are still being extensively investigated. Since the behavior and functionality of MSCs are tightly regulated by their surrounding niche, several cell priming/preconditioning approaches, including supplementation with bioactive molecules or chemicals, manipulation of oxygen tension, and employment of various culture conditions/substrates, have been proposed to improve the therapeutic efficiency of MSC transplantation [4].

Among the developed methods, we [5,6,7,8,9] and others [2,3,10,11,12] have reported that culturing MSCs in a three-dimensional (3D) multicellular spheroid configuration can significantly enhance both the viability of administered MSCs and their overall therapeutic functions. Specifically, the spheroid configuration can more closely recapitulate the physiological 3D microenvironment than conventional two-dimensional (2D) cultures [3]. Extensive cell–cell and cell–extracellular matrix (ECM) interactions can be established during MSC assembly and are well preserved throughout the whole procedure of cell administration, significantly superior to the trypsinized cell suspensions that are typically employed for cell therapy. Furthermore, a gradient of oxygen concentrations can be present within multicellular aggregates, thus generating a hypoxic niche that can effectively activate several signaling pathways, ultimately leading to the enhanced therapeutic potential of MSCs [13].

Nevertheless, most studies employ a variety of methods and parameters to engineer 3D MSC spheroids, and the resultant improvement in therapeutic capacity varies. Herein, we aim to manipulate the niche presented in cell spheroids by controlling the culture parameters. We believe that by characterizing the impact of the established artificial niches on multiple cellular behaviors, including the expression of paracrine factors, immunomodulatory activation, and metabolic switch, an optimal protocol for 3D MSC spheroid preparation for cell therapy can be developed, thus laying solid foundations for future advances and clinical applications of spheroid-based MSC therapy in various scenarios.

## 2. Materials and Methods

### 2.1. Cell Culture

MSCs that were derived from human umbilical cord blood and transfected with human telomerase reverse transcriptase (TERT) and red fluorescence protein (RFP) using nonviral vectors were acquired from the Bioresource Collection and Research Center, Food Industry Research and Development Institute, Hsinchu, Taiwan (Cat. No. BCRC 60605) and cultured according to the vendor’s protocol. The culture medium was composed of α-minimum essential medium (αMEM; Thermo Fisher Scientific, Waltham, MA, USA) containing 20% fetal bovine serum (Corning, Corning, NY, USA), 4 ng/mL basic fibroblast growth factor (PeproTech, Rocky Hill, NJ, USA), 30 µg/mL hygromycin B, and 200 µg/mL G418 sulfate (Thermo Fisher Scientific) [14]. For conventional monolayered cultivation, 1 × 10^6^ MSCs were seeded in a T75 flask and subcultured when they reach 90% confluence. All cells were cultivated at 37 °C in a humidified incubator supplemented with 5% carbon dioxide. For induction of cellular hypoxia, a humidified hypoxic incubator (1% oxygen; Heracell VIOS 160i, Thermo Fisher Scientific) was used for cell culture.

### 2.2. Preparation of 3D MSC Spheroids

The MSC spheroids were prepared using a methylcellulose (MC) hydrogel that was not bound by cells. Twelve percent (*w*/*v*) MC solution was prepared by dissolving MC powder in phosphate buffered saline (PBS; Thermo Fisher Scientific, St. Louis, MO, USA) [5,6,7,8,9]. After autoclaving, a sterilized MC solution was employed to coat each well of a 96-well culture plate. Before cell seeding, the MC solution-coated plate was pre-incubated at 37 °C for 30 min for hydrogel gelation. Next, MSCs were trypsinized, suspended in culture medium at the desired cell densities, transferred into MC hydrogel-coated plates, and cultivated for 24 h. The 3D MSC spheroids were imaged using a phase-contrast microscope (Olympus, Tokyo, Japan), and their diameters were determined using ImageJ software (National Institute of Health, Bethesda, MD, USA). Additionally, the viability of cells grown in spheroids was evaluated by staining with SYTOX Green (0.5 µM; Thermo Fisher Scientific) for 24 h before imaging using a confocal laser scanning microscope and analyzing using the ZEN blue software (Carl Zeiss, Oberkochen, Germany) [15]. Alternatively, the constructed MSC spheroids were enzymatically dissociated using trypsin, and the viability of the cells in the cell suspensions was determined using a trypan blue dye exclusion assay. For 2-deoxyglucose (2-DG) treatment, cell spheroids were treated with 5 mM 2-DG (Sigma-Aldrich, St. Louis, MO, USA) for 24 h prior to analysis [16].

### 2.3. Immunofluorescence Staining

For immunostaining, the 3D MSC spheroids were fixed in phosphate-buffered 4% paraformaldehyde for 30 min. After three washes with PBS, cell spheroids were treated with PBS supplemented with 0.5% Triton X-100 (Sigma-Aldrich) and 5% goat serum (Vector Laboratories, Burlingame, CA, USA) for 2 h for permeabilization and blocking before being incubated with primary antibodies against collagen type III, laminin, fibronectin, or heparan sulfate proteoglycan (HSPG; all from GeneTex, Hsinchu, Taiwan) at 4 °C overnight. The next day, the samples were washed with PBS five times and incubated with Alexa Flour 633-conjugated secondary antibody at 4 °C overnight. After five washes with PBS and counterstaining with Alexa Flour 488-conjugated phalloidin or DAPI (all from Thermo Fisher Scientific), the samples were mounted in a tissue-clearing solution (FocusClear solution, CelExplorer, Hsinchu, Taiwan) before being observed under a confocal laser scanning microscope.

### 2.4. Real-Time Quantitative Polymerase Chain Reaction (qPCR)

Total RNA of fifty cell spheroids was extracted using TRIzol Reagent (Thermo Fisher Scientific). After synthesis of complementary DNA with the High Capacity Reverse Transcription Kit, qPCR was conducted using Power SYBR Green PCR Master Mix with the StepOnePlus Real-Time PCR System (Thermo Fisher Scientific) [17]. The sequences of the primers used in the present study are listed in Table S1. The relative mRNA levels of target genes were determined by a comparative threshold cycle method and normalized to that of *RPL13A* [18,19,20]. For the control group, MSCs that were cultivated in a monolayer and had the same passage number and culture period as the MSC spheroids were used.

### 2.5. Statistical Analysis

All data are expressed as the mean ± standard deviation. Statistical analyses were performed using GraphPad Prism software (version 9.1; San Diego, CA, USA). One-way ANOVA with Tukey’s correction was employed for the comparison of three or more groups. Differences were considered significant at *p* < 0.05.

## 3. Results

### 3.1. MSCs Assemble into 3D Spheroids with Precise and Controllable Sizes Using MC Hydrogel-Coated Plates

**Figure 1:**
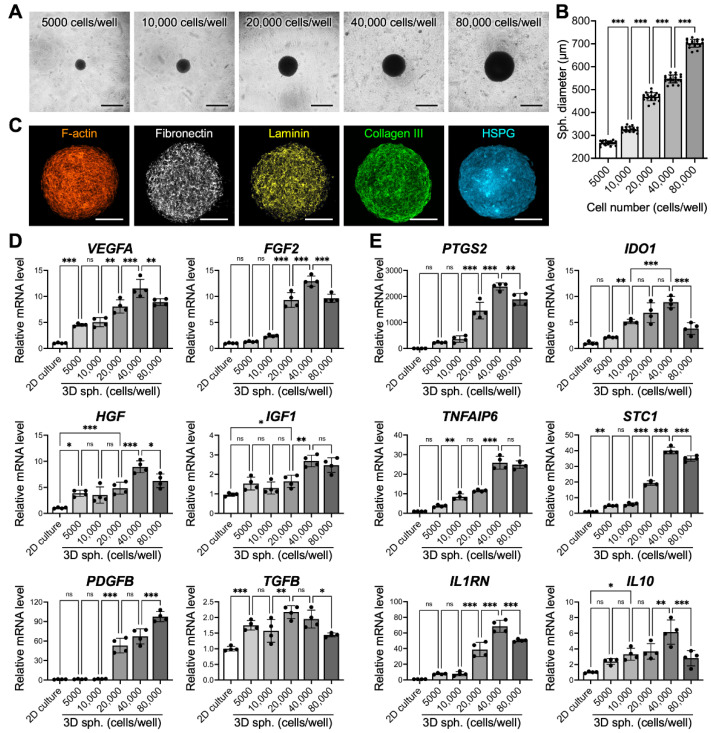
Mesenchymal stem cells (MSCs) assemble into three-dimensional (3D) spheroids (sph.) with precise and controllable sizes and exhibit enhanced therapeutic potential. (**A**) Representative images show the morphologies of 3D MSC spheroids fabricated using various cell seeding densities and (**B**) their corresponding diameters (*n* = 21 spheroids pooled from 3 independent experiments, mean ± s.d.) Scale bars, 500 µm. (**C**) Representative pseudocolored confocal images showing the organization of the cytoskeleton and the presence of extracellular matrix within the formed spheroids. Scale bars, 100 µm. The mRNA levels of genes that encode pro-regenerative (**D**) paracrine signaling molecules and (**E**) immunomodulatory enzymes/cytokines of MSCs grown with different 3D spheroid sizes or in two-dimensional (2D) monolayered culture are significantly different (*n* = 4). * *p* < 0.05; ** *p* < 0.01; *** *p* < 0.001; ns: not significant. HSPG: heparan sulfate proteoglycan.

We first investigated the effects of cell seeding densities on the sizes of the formed spheroids. After culture in MC hydrogel-coated plates for 24 h, MSCs assembled into a single 3D spheroid in each well (Figure 1A). The diameters of the thus-formed 3D spheroids that were prepared with cell seeding densities of 5000, 10,000, 20,000, 40,000 and 80,000 cells per well were 266.9 ± 9.1, 326.2 ± 10.6, 469.1 ±17.7, 547.1 ± 20.5, and 702.7 ± 26.2, respectively (Figure 1B), suggesting that the spheroid sizes can be precisely manipulated by tuning the cell seeding densities. The assembled 3D MSC spheroids were also harvested and processed to characterize their ECM content by immunostaining. Figure 1C shows representative confocal images of matrix proteins in the equatorial plane (the widest cross-section) of the spheroids, demonstrating their abundance of multiple ECM components, which are crucial for MSC survival and functionality.

### 3.2. Spheroid Size Impacts MSC Paracrine and Immunomodulatory Potential

The mRNA levels of various paracrine factors (*VEGFA*, *FGF2*, *HGF*, *IGF1*, *PDGFB*, and *TGFB*) or immunomodulatory enzymes (*PTGS2* and *IDO1*) and factors (*TNFAIP6*, *STC1*, *IL1RN* and *IL10*) in the 3D MSC spheroids fabricated with different cell seeding densities were determined by qPCR. Overall, the 3D MSC spheroids displayed significantly increased expression of the investigated genes compared with that of the MSCs that were cultivated under conventional 2D conditions (Figure 1D,E; *p* < 0.001), in agreement with the literature [2,3,21]. Furthermore, the degree of enhancement was highly correlated with the size of the spheroids, a phenomenon that has been reported previously [2]. For most of the investigated paracrine factors and all of the immunomodulatory genes, the highest upregulation of mRNA expression was found in the 3D MSC spheroids prepared by 40,000 cells per well. For example, the mRNA levels of *VEGFA*, *FGF2* and *PDGFB*, which are critical for vessel formation and stabilization, in 3D MSC spheroids fabricated using 40,000 cells showed 2.6-, 10.5- and 34.2-fold increases, respectively, compared to those prepared using 5000 cells (Figure 1D; *p* < 0.001). A similar trend was also observed in the expression of pro-survival factors (2.3- and 1.8-fold increases in the mRNA levels of *HGF* and *IGF1*, respectively; Figure 1D, *p* < 0.001), immunomodulatory enzymes (10.3- and 4.2-fold increases in the mRNA levels of *PTGS2* and *IDO1*, respectively; Figure 1E, *p* < 0.001) and factors (6.9-, 8.4-, 8,8- and 2.6-fold increases in the mRNA levels of *TNFAIP6*, *STC1*, *IL1RN* and *IL10*, respectively; Figure 1E, *p* < 0.001) when comparing 3D MSC spheroids that were assembled by 40,000 cells with those assembled by 5000 cells. Therefore, spheroids prepared from 40,000 MSCs were chosen for the following studies.

### 3.3. The Culture Period also Affects MSC Viability and Therapeutic Potential

**Figure 2:**
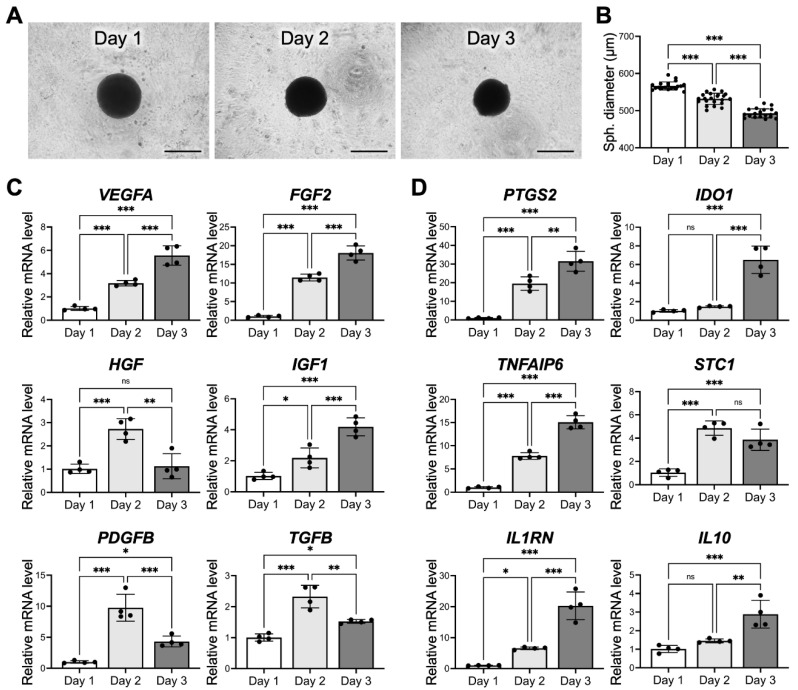
The culture period of 3D MSC spheroids affects their therapeutic potential. (**A**) Representative images show the morphologies of 3D MSC spheroids cultivated for various durations and (**B**) their corresponding diameters (*n* = 21 spheroids pooled from 3 independent experiments, mean ± s.d.). Scale bars, 500 µm. The expression levels of multiple pro-regenerative genes that encode (**C**) paracrine signaling molecules and (**D**) immunomodulatory enzymes/cytokines by MSCs (*n* = 4). * *p* < 0.05; ** *p* < 0.01; *** *p* < 0.001; ns: not significant.

We then next evaluated whether the duration of spheroid culture also plays a role in modulating the expression profiles of therapeutic genes. Figure 2A shows representative images of 3D MSC spheroids assembled by 40,000 cells and incubated for 1, 2 or 3 days. The average diameters of the spheroids that were cultivated for 1, 2, and 3 days were 556.5 ± 15.8, 525.3 ± 15.1, and 462.7 ± 12.2 µm, respectively (Figure 2B), suggesting that the spheroid size decreased gradually as time progressed. For paracrine and immunomodulatory genes, the expression levels of *VEGFA*, *FGF2*, *IGF1*, *PTGS2*, *IDO1*, *TNFAIP6*, *STC1*, *IL1RN* and *IL10* were elevated with time (Figure 2C). However, the mRNA levels of *HGF*, *PDGFB* and *TGFB* peaked on day 2 (Figure 2D).

**Figure 3:**
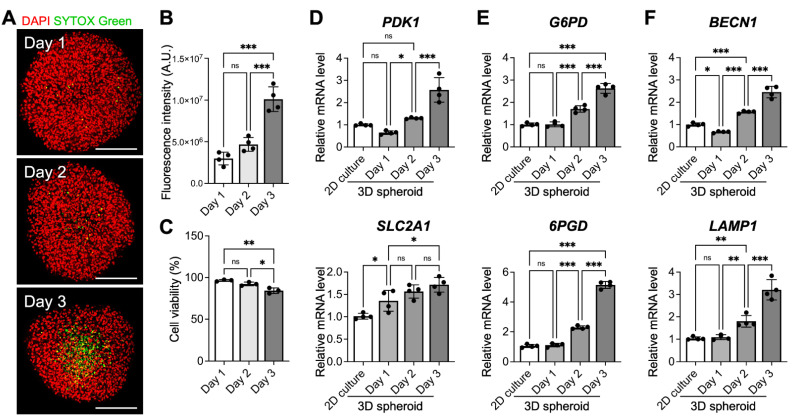
Prolonged cultivation of 3D MSC spheroids leads to cell death in the spheroid core and activates metabolic reconfiguration and autophagy. (**A**) Representative pseudocolored confocal images show dead SYTOX Green-positive cells and (**B**) the corresponding fluorescence intensities of SYTOX Green in the equatorial plane of the spheroids (*n* = 4). Scale bars, 200 µm. (**C**) The viability of MSC grown in 3D cell spheroids was determined using a trypan blue dye exclusion assay (*n* = 3). The expression levels of genes that are associated with (**D**) glycolysis, (**E**) the pentose phosphate pathway and (**F**) autophagy were investigated (*n* = 4). * *p* < 0.05; ** *p* < 0.01; *** *p* < 0.001; ns: not significant.

It is presumed that incubating 3D cell spheroids with a radius exceeding the distance of oxygen diffusion limitation, typically 250 µm, for a prolonged period can lead to significant cell death in the interior of spheroids [22,23]. To investigate the viability of MSCs within the grown spheroids, we used SYTOX Green, a membrane impermeable dye, to label the dead cells. As revealed in the representative confocal images in Figure 3A, only a few SYTOX Green-positive nuclei could be detected at the equatorial plane of the spheroids incubated for 1 and 2 days. On day 3, however, a remarkable increase in the number of dead cells was observed in the interior of the spheroid (Figure 3A,B), suggesting the development of a necrotic core. The results of trypan blue dye exclusion assay also confirmed the decreased cell viability of the cells grown in 3D cell spheroids for 3 days (Figure 3C). To ensure that the majority of the MSCs remained viable before being employed for cell therapy, cell spheroids that were prepared with a 2-day incubation were used for subsequent investigations.

### 3.4. Three-Dimensional MSC Spheroids Exhibit Signs of Metabolic Reconfiguration and Enhanced Autophagy

We also assessed the gene expression profile of some metabolic enzymes that are involved in glycolysis (*PDK1* and *SLC2A1*) and the pentose phosphate pathway (*G6PD* and *6PGD*). The qPCR results displayed in Figure 3D,E indicate that the mRNA levels of all investigated genes increased as the incubation time increased (*p* < 0.05), suggesting a gradually elevated dependence on glycolysis and the pentose phosphate pathway for ATP production. Moreover, the expression levels of *BECN1* and *LAMP1*, autophagy-related genes, were enhanced significantly in the 3D MSC spheroids after a 3-day culture (Figure 3F; *p* < 0.001), indicating the activation of autophagy in MSCs grown in cell spheroids.

### 3.5. The Enhanced Therapeutic Potential of MSCs after Assembly into a 3D Spheroid Configuration Is Not Fully Attributed to the Development of a Hypoxic Niche

**Figure 4:**
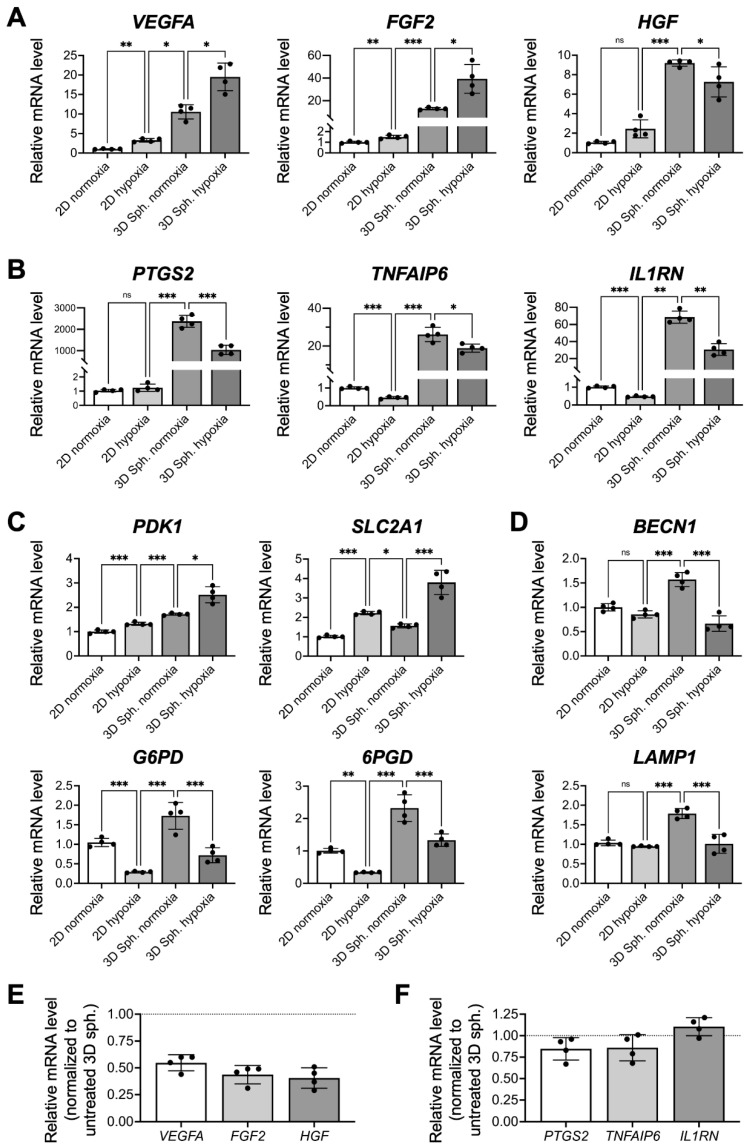
The enhanced therapeutic potential of MSCs after assembly into a 3D spheroid configuration is not fully attributed to the development of a hypoxic niche. The mRNA levels of genes that encode (**A**) paracrine signaling molecules, (**B**) immunomodulatory enzymes/cytokines, (**C**) metabolic enzymes/transporters and (**D**) autophagic markers in MSCs grown in 2D or 3D configurations under normoxic or hypoxic conditions were explored (*n* = 4). (**E**) Relative gene expression of paracrine signaling molecules or (**F**) immunomodulatory enzymes/cytokines after glycolytic inhibition using 5 mM 2-deoxyglucose (*n* = 4). * *p* < 0.05; ** *p* < 0.01; *** *p* < 0.001; ns: not significant.

To investigate whether the hypoxic niche is the major factor that contributes to the enhanced therapeutic potential of MSCs, we compared the mRNA expression profiles of multiple genes in 2D-cultivated cells and 3D cell spheroids that were grown under normoxic (21% O_2_) or hypoxic (1% O_2_) conditions. MSCs grown under hypoxia displayed significant upregulation of pro-angiogenic *VEGFA* and *FGF2* genes and pro-survival *HGF* gene compared to MSCs cultivated under normoxic conditions (3.3-, 1.5- and 2.4-fold, respectively; Figure 4A), suggesting that a hypoxic niche could be used to enhance the expression of these paracrine factors. However, the elevated mRNA levels of the paracrine factors in cells grown under 2D hypoxic conditions remained far from those of the 3D MSC spheroids that were assembled under normoxic conditions, suggesting that other niche factors in addition to the naturally developed hypoxic core also contributed significantly to the highly increased expression of therapeutic factors in 3D spheroids. Moreover, the expression of *VEGFA* and *FGF2* could be further upregulated once the spheroids were incubated under hypoxic conditions (1.8- and 3.1-fold increase, respectively; Figure 4A; *p* < 0.05), indicating that the development of the hypoxic core in 3D MSC spheroids grown under normoxia was rather limited.

For the expression of immunomodulatory enzymes and cytokines, the hypoxic niche induced a dramatically different outcome compared with that of paracrine factors. As shown by the qPCR results in Figure 4B, MSCs cultivated under 2D hypoxic conditions showed decreased mRNA levels of *TNFAIP6* and *IL1RN* when compared to those grown in normoxic conditions (*p* < 0.001), suggesting that the hypoxic niche might have a deleterious effect on the immunomodulatory potential of MSCs. Moreover, incubation of 3D MSC spheroids under hypoxia led to a substantial decrease in mRNA expression of the investigated genes (Figure 4B, *p* < 0.05), indicating that cellular hypoxia could indeed impair the immunomodulatory capacity of MSCs.

We then further investigated the modulation of metabolic genes of MSCs. Upon exposure to hypoxia, the 2D-cultivated MSCs showed significantly upregulated expression of *PDK1* and *SLC2A1* and downregulated expression of *G6PD* and *6PGD* (Figure 4C, *p* < 0.01), a clear indication of MSC metabolic remodeling toward glycolysis with a reduced pentose phosphate pathway. The observed metabolic shift was consistent with the findings reported in the literature [24,25]. For 3D MSC spheroids prepared under normoxia, however, changes in the mRNA levels were rather limited, while hypoxia-cultivated 3D MSC spheroids displayed a remarkable shift in the expression level of the investigated genes (Figure 4C, *p* < 0.05). Moreover, the observed upregulation of autophagy-related genes in 3D MSC spheroids was abolished when exposed to hypoxic conditions (Figure 4D; *p* < 0.001), suggesting the deactivation of autophagy in MSCs grown in hypoxic cell spheroids.

To elucidate whether the observed metabolic shift was crucial for the therapeutic functions of 3D MSC spheroids, the glycolytic inhibitor 2-DG was used to treat cell spheroids. As revealed in the qPCR results, a dramatic down-regulation of *VEGFA*, *FGF2*, and *HGF* genes was detected (Figure 4E), while the expression of immunomodulatory genes remained unchanged (Figure 4F). These results demonstrated that the slight metabolic switch observed after spheroid assembly might have a significant impact on the paracrine potential of 3D MSC spheroids.

## 4. Discussion

While MSC-based cell therapies have shown promise for treating various diseases and injuries, strategies that can enhance the overall effectiveness and reduce the observed therapeutic variability are urgently needed. By engineering a native tissue-mimicking 3D microenvironment, researchers have shown that MSC spheroids possess an elevated pro-regenerative ability [3,11]. The present study attempted to elucidate the relationship between the cell seeding density and thus the size of 3D MSC spheroids, the incubation period, and the resultant alternations in their paracrine/immunomodulatory potential for the purpose of optimizing parameters for the preparation of 3D MSC spheroids for cell therapy.

Since the in vivo therapeutic potential of RFP- and TERT-transfected MSCs has been previously verified using various animal models [5,6,7,8], we believe that these MSCs are suitable for use in the present study for further investigation. For a determination of the effect of spheroid size on therapeutic potential, a method that can fabricate spheroids with the desired diameters must be used. Herein, by controlling the number of cells seeded into each well of MC hydrogel-coated plates [5,6,7,8,9], we could precisely manipulate the size of the formed spheroids, which is crucial for ensuring the consistency of the following therapeutic effect after cell transplantation. The harvested MSC spheroids contained abundant matrix components, including fibronectin, laminin, collagen, and HSPG. In addition to providing physical support for cell adhesion, these matrix proteins are known to regulate multiple cellular behaviors, including the migration, proliferation and differentiation of MSCs, via outside-in signaling [1,26,27]. Furthermore, a close correlation between the secretory activity of MSCs and their surrounding matrixes has been extensively reported in recent studies [28,29,30,31], suggesting that the niche established by the ECM can be critical for the therapeutic function of MSCs. Moreover, HSPGs are known to act as depots to entrap various soluble factors and to protect their bioactivity [32]. Therefore, the presence of HSPG indicated that the growth factors or cytokines released by MSCs during in vitro cultivation can be retained within spheroids during transplantation and thus may have direct actions on both the delivered cells and the target tissues.

MSCs are known to execute therapeutic functions by releasing pro-angiogenic vascular endothelial growth factors and basic fibroblast growth factors, pro-survival hepatocyte growth factors and insulin-like growth factor-1, mitogenic platelet-derived growth factors, and the multifunctional cytokine transforming growth factor-β [33,34]. Additionally, MSCs can modulate local inflammation via (1) expression of prostaglandin-endoperoxide synthase (also known as cyclooxygenase) 2 and indoleamine 2,3-dioxygenase-1, which can produce prostaglandin E2 and kynurenine, respectively [35], or (2) direct secretion of tumor necrosis factor-inducible gene 6 protein, stanniocalcin-1, interleukin (IL)-1 receptor antagonist, and IL-10, which regulate the activity of immune cells or cytokines [36,37,38].

In agreement with the literature [2,3,21], after assembly into the 3D spheroid configuration, MSCs were self-activated and exhibited significantly enhanced therapeutic potential in terms of paracrine signaling and immunomodulatory activity when compared to those grown in conventional 2D monolayered culture, demonstrating the importance of the 3D niche for promoting the therapeutic capacity of MSCs. Furthermore, our results indicated that the characteristics of 3D MSC spheroids were crucially dependent on the experimental parameters. In the present study, MSC spheroids that were assembled by 40,000 cells and cultivated for 2 days with a diameter of approximately 525 µm showed the best therapeutic potential. The discrimination between the investigated groups was thought to be attributed to the difference in the intrinsic gradients of oxygen, nutrients and signal molecules present in the spheroids [39,40]. Typically, as the spheroid size increases, the limited availability of oxygen owing to the increased diffusion distance is considered to mainly account for the development of a hypoxic core in spheroids, thereby altering cellular behaviors or even causing the death of interior cells [41,42,43]. In the present study, the largest MSC spheroids that were assembled by 80,000 cells and had a diameter of approximately 700 µm displayed, decreased mRNA expression of multiple therapeutic proteins, a finding that is consistent with a previous investigation. Furthermore, for MSC spheroids that were assembled by 40,000 cells, although the cell viability was similar on the first two days, a significantly increased cell death was observed in the center of the spheroid after a three-day culture, which could be attributed to prolonged hypoxia.

In addition to hypoxia, other local niches established within the 3D MSC spheroids might also contribute significantly to the altered cellular behaviors and therapeutic potential. In fact, recent literature has reported that the reduction of oxygen tension within spheroids is not as high as originally thought [40,44]. Compared to that of MSCs exposed to hypoxic conditions, the upregulated expression of the *PDK1* and *SLC2A1* genes, which encode a key glycolytic enzyme (pyruvate dehydrogenase kinase 1) and transporter (glucose transporter 1), respectively, in assembled cell spheroids was limited, suggesting that the activation of glycolysis-related genes by hypoxia-inducible factor-1α could be minimal. Furthermore, hypoxic MSCs showed reduced expression of *G6PD* (encodes glucose-6-phosphate dehydrogenase) and *6PGD* (6-phosphogluconate dehydrogenase), the two enzymes involved in the pentose phosphate pathway, which is consistent with findings reported in the literature [24]. Conversely, the expression of *G6PD* and *6PGD* was upregulated as the incubation time increased, indicating the effect of other niche factors, instead of hypoxia, inherent in the spheroids on the modulation of cellular metabolism. As an enhancement of the pentose phosphate pathway is reportedly associated with MSC survival and paracrine functionality [25,45], we speculated that metabolic reconfiguration upon spheroid assembly could be an important niche factor contributing to the enhanced therapeutic potential of MSCs.

Despite the success in optimizing parameters for the fabrication of 3D MSC spheroids with superior paracrine and immunomodulatory potential, several limitations still need to be addressed prior to future application in clinical scenarios. First, cell spheroids prepared using different approaches have heterogeneous characteristics, thus resulting in variations in therapeutic potential. For example, with an identical cell seeding density, spheroids fabricated via the hanging drop method, using ultralow attachment plates, or employing MC hydrogels, showed different spheroid sizes, morphologies, and cell packing densities [11,39]. Therefore, an approach that can constantly generate uniform cell spheroids is needed before applying the established protocols and parameters. Second, the present study only analyzed the overall gene expression of MSC spheroids, which contained heterogeneous populations of cells exposed to various niches and should exhibit different profiles. Technologies such as single-cell sequencing that can distinguish multiple cell populations and provide a higher resolution of cellular differences should be employed to elucidate the mechanism underlying the observed beneficial effects. Finally, protein-level-based analyses for animal investigations, which were not used in the current study, are necessary to verify the metabolic shift and the ultimate therapeutic efficacy of the optimized 3D MSC spheroids. It is worth noting that the MSCs used herein were non-virally transfected with RFP and TERT [14]. Although maintenance of the differentiation capacities of transfected MSCs has been demonstrated [14], further investigations are needed to verify the effects of transfection on the improved therapeutic potential observed in the present study.

## 5. Conclusions

In summary, we demonstrated that by optimizing the cultivation parameters of 3D MSC spheroids, the inherent niches could be well tuned to activate the expression of several critical pro-regenerative paracrine molecules and immunomodulatory factors, thereby further potentiating the therapeutic capacity prior to cell transplantation. We expect that our findings can serve as guidelines for the future design of 3D MSC spheroids for cell-based therapies by providing a new insight into the mechanisms for the enhanced therapeutic benefits.

## Data Availability

Data are available from C.-C.H. upon request.

## Supplementary Materials

The following are available online at https://www.mdpi.com/article/10.3390/cells10102747/s1, Table S1: Primer sequence used for real-time quantitative polymerase chain reaction.
