# Supplementary material for: Modulation of Inherent Niches in 3D Multicellular MSC Spheroids Reconfigures Metabolism and Enhances Therapeutic Potential
Source: Cells. 2021 Oct 14;10(10):2747. doi: 10.3390/cells10102747 (PMC8534378; doi:10.3390/cells10102747)
Supplement: Supplementary file 1 [file cells-10-02747-s001.zip › cells-1394860-supplementary.pdf]

Table S1. Primer sequence used for real-time quantitative polymerase chain reaction.

Gene	Forward	Reverse
6PGD	5'-ATTCTCAAGTTCCAAGACACCG-3'	5'-GTGGTAAAACAGGGCATGGGA-3'
BECN1	5'-TGTCCACAGAAAGTGCCAACA-3'	5'-CCTCA CAGAGTGGGTGATCCA-3'
FGF2	5'-AGCGGCTGTACTGCAAAAAC-3'	5'-GCTTGAAGTTGTAGCTTGATGTG-3'
G6PD	5'-TGAGCCAGATAGGCTGGAA-3'	5'-TAACGCAGGCGATGTTGTC-3'
HGF	5'-AAGGTGACTCTGAATGAGTC-3'	5'-GGCACATCCACGACCAGGAACA-3'
IDO1	5'-GCCCTTCAAGTGTTTCACCAA-3'	5'-GCCTTTCCAGCCAGACAAATAT-3'
IGF1	5'-GGTGGATGCTCTTCAGTTCGTG-3'	5'-AAATGTACTTCCTTCTGGGTCTT-3'
IL1RN	5'-AAGATGTGCCTGTCCTGTGTCAA-3'	5'-GTTCTCGCTCAGGTCAGTGATGTTA-3'
IL10	5'-GACTTTAAGGGTTACCTGGGTTG-3'	5'-TCACATGCGCCTTGATGTCTG-3'
LAMP1	5'-CAGATGTGTTAGTGGCACCCA-3'	5'-TTGGAAAGGTACGCCTGGATG-3'
PDGFB	5'-GGCCGAGTTGGACCTGAACATG-3'	5'-GAAGTTGGCGTTGGTGCGGTCTA-3'
PDK1	5'-GGAACAGCGCAGTACGTTTCT-3'	5'-CTCGTTTCCAGCTCGGAATGG-3'
PTGS2	5'-GAATGGGGTGATGAGCAGTT-3'	5'-CAGAAGGGCAGGATACAGC-3'
RPL13A	5'-CATAGGAAGCTGGGAGCAAG-3'	5'- GCCCTCCAATCAGTCTTCTG-3'
TGFB	5'-CCCAGCATCTGCAAAGCTC-3'	5'-GTCAATGTACAGCTGCCGCA-3'
TNFAIP6	5'-GATGGATGGCTAAGGGCAGAGT-3'	5'-TCATTTGGGAAGCCTGGAGATT-3'
VEGFA	5'-TCTTCAAGCCATCCTGTGTG-3'	5'-ATCCGCATAATCTGCATGGT-3'
SLC2A1	5'-CTTCACTGTCGTGTCGCTGT-3'	5'-TGAAGAGTTCAGCCACGATG-3'
STC1	5'-TAGGTTTTTGGCTGAGTACTGGAA-3'	5'-ACCTTTATATGCACATGGAGCTTA-3'
